# Decoding the Unseen: A Systematic Review of the Analytical Performance of Nitazene Test Strips for Identifying Synthetic Opioids in Seized Drug Materials

**DOI:** 10.7759/cureus.99109

**Published:** 2025-12-13

**Authors:** Abdulkreem Al-Juhani, Abdulelah Alasmari, Omar O Aljohani, Hatoon Hakeem, Rodan Desoky, Muhannad A Badghaish, Naif Aljohani, Fai A Alanazi

**Affiliations:** 1 Forensic Medicine, Forensic Medicine Center, Jeddah, SAU; 2 Surgery, King Abdulaziz University Faculty of Medicine, Jeddah, SAU; 3 Forensic Medicine, Ministry of Health, Jeddah, SAU; 4 Forensic Medicine, Ministry of Health, Riyadh, SAU; 5 Emergency Department, Prince Mohammed Bin Abdulaziz Hospital, Madinah, SAU; 6 Medicine, Alfaisal University College of Medicine, Riyadh, SAU; 7 Forensic Medicine, Forensic Center Jeddah, Jeddah, SAU; 8 Forensic Medicine, Riyadh Forensic Medicine Center, Riyadh, SAU; 9 Forensic Science (Forensic Genetics and Molecular Biology), Naif Arab University for Security Sciences, Riyadh, SAU

**Keywords:** forensic medicine, forensics, nitrazine test, test strips, toxicology

## Abstract

Nitazenes are highly potent synthetic opioids increasingly detected in illicit drug markets and associated with significant public-health and forensic challenges. Rapid identification tools, such as lateral-flow immunoassay (LFA) test strips, are widely used in harm-reduction and forensic settings; however, their analytical performance for detecting nitazene-class opioids remains insufficiently characterized. This systematic review aims to evaluate the analytical performance of nitazene LFA test strips when used on seized drug materials and laboratory-prepared solutions, compared with mass spectrometry (MS) reference methods (liquid chromatography-tandem mass spectrometry (LC-MS/MS), liquid chromatography-quadrupole time-of-flight mass spectrometry (LC-QTOF-MS), and gas chromatography-mass spectrometry (GC-MS)). Outcomes included sensitivity, specificity, cross-reactivity, limits of detection, and operational interferences. Adhering to the Preferred Reporting Items for Systematic Reviews and Meta-Analyses - Diagnostic-Test Accuracy (PRISMA-DTA) guidelines and applying Quality Assessment of Diagnostic Accuracy Studies-2 (QUADAS-2) framework, five eligible studies were synthesized, including laboratory evaluations and field-paired analyses. Only studies evaluating LFAs on drug materials - not biological samples - were included. Our results showed that two studies provided paired LFA-mass-spectrometry datasets. Under concentrated preparation conditions (10 mg/1 mL), LFAs demonstrated complete agreement with MS. Sensitivity declined at higher dilution (10 mg/5 mL), while specificity remained high. Analytical detectability varied substantially across nitazene analogues, with N-desethyl-metonitazene exhibiting the greatest sensitivity. Matrix effects, caffeine adulteration (~300 µg/mL), solvent concentrations >10% acetonitrile, and elevated temperatures all reduced line intensity or hindered wicking.

In conclusion, Nitazene LFA test strips show potential value as preliminary material-based screening tools in forensic and harm-reduction applications, but should not be interpreted as confirmatory. Their use requires standardized protocols, conservative interpretation rules, and mandatory mass-spectrometric confirmation. Large, independent, multi-site analytical validation studies are needed to establish reliability, optimize field use, and support integration into drug-checking programs.

## Introduction and background

New synthetic opioids from the nitazene class have quickly appeared in the illegal drug trade. This makes things very hard for forensic labs, drug-checking services, and public health monitoring programs [1,2]. These molecules generated from benzimidazole demonstrate exceptional potency, frequently surpassing that of fentanyl, and persist in powders, counterfeit tablets, and paraphernalia residues linked to both fatal and non-fatal intoxications [3]. It is hard to find nitazenes on a regular basis since they have different structures, and it is easy to produce new analogues. This is especially true because many nitazenes are not included in typical opioid immunoassays [4]. 

Lateral-flow immunoassays (LFAs), often called rapid test strips, are becoming more popular for preliminary screening of illegal substances in harm-reduction and forensic settings because they are cheap, easy to carry, and give quick visual results [5]. 

However, the LFA products that are currently available were originally designed for regular opioids, and their antibodies may not work well with the chemically different nitazene family. This raises issues about false-negative and false-positive results, especially when the strips are used on complicated street-level powders that have more than one adulterant and excipient [6, 7]. To guarantee dependable substance identification, the analytical efficacy of nitazene LFAs must be assessed against high-specificity confirmatory techniques, including liquid chromatography-tandem mass spectrometry (LC-MS/MS) and gas chromatography-mass spectrometry (GC-MS), which are regarded as the standard methodologies in forensic toxicology [8, 9]. However, current research exhibits significant variability in sample preparation, analogue makeup, dilution techniques, ambient conditions, and interpretation guidelines, hindering the capacity to draw conclusive determinations regarding their trustworthiness [10,11,12]. 

This systematic review consolidates the existing information about the analytical efficacy of nitazene lateral-flow test strips when utilized on confiscated illicit substances or laboratory-synthesized powders, employing MS-based methodologies as the benchmark standard. The review analyzes sensitivity, specificity, analogue-level detectability, cross-reactivity, matrix interferences, and operational factors pertinent to forensic and harm-reduction practices. The goal is to make it clear what nitrazine LFAs can and can't do as preliminary material-based screening methods and to find important gaps that need more analytical validation.

## Review

Methodology 

Study Design and Search Strategy 

This systematic review was conducted in accordance with the PRISMA-DTA framework, with the “target condition” defined as the presence of nitazene-class synthetic opioids in seized drug materials or laboratory-prepared powders, rather than any clinical or diagnostic condition. The index tests were lateral-flow immunoassay (LFA) strips intended for nitazene or non-fentanyl opioid detection, and the reference standards were mass-spectrometric methods, including LC-MS/MS, liquid chromatography-quadrupole time-of-flight mass spectrometry (LC-QTOF-MS), high-resolution mass spectrometry (HRMS), and GC-MS. A thorough search was conducted in the following databases: PubMed/MEDLINE, Embase, Scopus, Web of Science, and Google Scholar, encompassing papers from November 2015 to November 2025. The search was enhanced by gray literature sources, such as ChemRxiv, medRxiv, Center for Forensic Science Research and Education (CFSRE)/Novel Psychoactive Substances (NPS) Discovery, DrugsData, Institute of Environmental Science and Research (ESR) New Zealand analytical reports, and manufacturer white papers. Search queries included both restricted vocabulary and free-text words about nitazenes, synthetic opioids, immunoassay testing, and analytical validation. An example of a search string is: (nitazene OR benzimidazole opioid OR isotonitazene OR metonitazene OR protonitazene OR "non-fentanyl opioid*") AND (lateral flow OR immunoassay OR "test strip*" OR LFA) AND (analytical accuracy OR sensitivity OR specificity OR "limit* of detection") AND (LC-MS OR LC-MS/MS OR GC-MS OR HRMS). We manually went through the reference lists of all the studies that were included to find more records. Duplicate records were removed in EndNote (Clarivate Plc, London, UK), followed by additional de-duplication and title/abstract screening in Covidence (Covidence, Melbourne, Australia). No machine-learning-based exclusion tools were used; all automated decisions were manually verified.

Eligibility Criteria 

Studies were eligible for inclusion if they met all the following criteria: evaluated a lateral-flow immunoassay designed to detect nitazenes or related non-fentanyl synthetic opioids. Tested seized materials, drug powders, tablet residues, or prepared solutions (biological samples were excluded). Employed mass spectrometry as a reference standard or provided sufficient analytical validation data (e.g., limit of detection (LOD), cross-reactivity). Reported outcomes related to sensitivity, specificity, signal intensity, LOD, cross-reactivity, repeatability, or interference effects. Our exclusion criterion was to exclude editorials, commentaries, opinion pieces, animal studies, studies lacking reproducible methodological description, and studies addressing only fentanyl or non-nitazene opioids without nitazene data.

Study Selection

**Figure 1:**
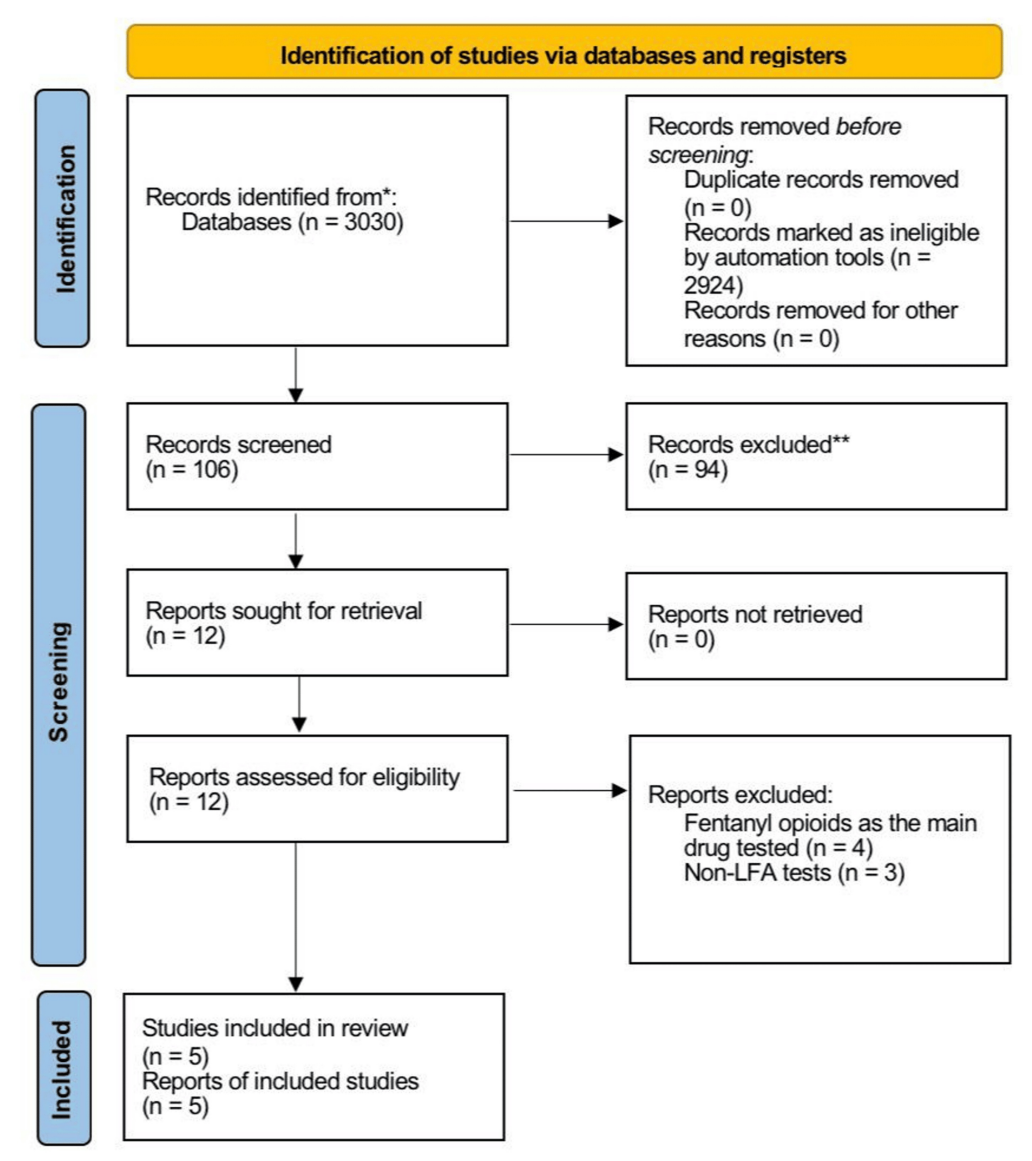
PRISMA Flow Diagram PRISMA 2020 flow diagram for new systematic reviews, which included searches of databases and registers only. LFA: lateral-flow immunoassay

Two reviewers independently screened titles, abstracts, and full texts. Discrepancies were resolved by discussion and consensus. Automated filtering tools were used to remove duplicate digital records, and all automated exclusions were subsequently verified by manual screening to ensure accuracy. A PRISMA flow diagram (Figure 1) was constructed to document all stages of search, screening, and inclusion. All included studies evaluated LFAs on powders, seized materials, or laboratory-prepared solutions; none evaluated clinical specimens.

Data Extraction

Data were extracted using a structured template that captured key methodological and analytical variables from each study, including the type of nitazene analogue tested, the LFA brand and lot number, sample preparation methods, dilution ratios, cut-off thresholds, and interpretation rules. Information related to environmental and operational conditions - such as temperature, solvent composition, and potential interference - was also recorded. For studies that employed mass-spectrometric confirmation, details of the reference method and timing were extracted, and paired results were used to reconstruct true-positive, false-positive, false-negative, and true-negative outcomes when available. Additional analytical parameters, including limits of detection, cross-reactivity, and lot-to-lot variability, were collected to support narrative synthesis. All extracted data were independently reviewed by two investigators to ensure accuracy and consistency.

Quality Assessment, Data Synthesis, and Analysis 

Methodological quality was evaluated using the QUADAS-2 tool, tailored for material-based analytical testing. The domains assessed included: Sample selection, Execution of the index test, Execution of the reference standard, Flow and timing, and Funding or manufacturer involvement. Studies lacking MS confirmation for negative samples or involving manufacturer funding were rated as high risk. Because complete paired datasets were available from only two studies, a narrative structured synthesis was performed. Sensitivity and specificity were calculated with Clopper-Pearson exact 95% confidence intervals for paired studies. Quantitative pooling through bivariate random-effects meta-analysis was planned but not conducted due to insufficient data. Analytical characteristics - including LOD, cross-reactivity, solvent effects, excipient interference (e.g., caffeine), lot variability, and temperature-based changes - were summarized descriptively across studies.

Results

Study Selection and Characteristics

Five papers passed the inclusion criteria and were used in the final synthesis: De Vrieze (2024) [1], Marland (2025) [2], BTNX/Chicago Recovery Alliance (CRA) White Paper (2024) [3], ESR NZ (2024) [4], and Edward Sisco (2024) [5]. These studies combined laboratory evaluations, bench validation panels, and field-based diagnostic assessments of nitazene lateral-flow immunoassay (LFA) test strips. Table 1 displays research conducted in academia and manufacturer-affiliated settings, utilizing the BTNX Rapid Response™ (BTNX Inc., Pickering, Canada) brand for all tests. Two studies (De Vrieze 2024 [1] and BTNX/CRA 2024 [3]) explicitly compared LFA results to a mass-spectrometry reference (LC-QTOF-MS or GC/MS), but the other three (Marland 2025 [2], ESR NZ 2024 [4], and Sisco 2024 [5]) focused on analytical performance, cross-reactivity, and interferences without diagnostic pairing. All tests used the manufacturer's competitive immunoassay rule (absence of test line = positive), with read times ranging from 5 to 10 minutes. Lot numbers varied between experiments, indicating possible lot-to-lot variability (Table 1).

**Table 1:** Study characteristics and index-test conduct All LFAs are competitive (absence of test line = positive). Read time ≤ 10 min per manufacturer's instructions. Flow and timing indicate the interval between the strip test and MS confirmation. BTNX Rapid Response™ by BTNX Inc., Pickering, Canada. ImageJ is free software for basic scientific image analysis (https://imagej.net/ij/). Abbreviations: LFA = lateral-flow assay; LOD = limit of detection; MS = mass spectrometry; LC-QTOF-MS = liquid chromatography-quadrupole time-of-flight MS; COI = conflict of interest; CFSRE = Center for Forensic Science Research and Education; GC = gas chromatography; TP = true positive; lot A/B = lot A and lot B

Study (year)	Setting/sample type	Brand/lot	Funding/conflicts	Read time & positivity rule	Reader blinding/method	Sample preparation/dilution	Flow & timing vs MS	Reference standard	Notes
De Vrieze 2024 [1]	Laboratory + 6 authentic powders	BTNX Rapid Response™ Lots A/B	Independent academic (no COI)	Competitive LFA (“absence of T line = positive”); 5 & 10 min	2 visual + ImageJ (blinded)	Water suspension; some undissolved	Same sample tested ≤ 1 h by LC-QTOF	LC-QTOF-MS	6/6 authentic TP; no negatives.
Marland 2025 [2]	Lab; 36 nitazenes, 93 adulterants, 3 seized heroin	BTNX lots DOAB24050015/DOAB24020005	Academic (no COI)	≤ 10 min; competitive	Dual visual readers	Tap water; LOD by analogue	Same-day triplicates (no MS)	—	Caffeine fading (~300 µg/mL); LOD 250 ng–100 µg/mL.
BTNX/CRA 2024 White Paper [3]	Field; street samples (USA)	BTNX Rapid Response™	Manufacturer sponsored	Competitive; faint line = negative	Field visual readers	10 mg / 1 mL and 10 mg / 5 mL	Strip then GC/MS ≤ 24 h	GC/MS (DrugsData)	n = 11 paired; prelim sens 100 / 87.5%.
ESR NZ 2024 [4]	Service lab; drug checking (NZ)	BTNX lot DOAB23120001	Govt funded (no COI)	Dip 15 s; read ≤ 10 min	Visual single reader	1 mL water; serial dilutions	Same sample; no MS	—	Poor solubility for etonitazene noted.
Sisco (2024) [5]	Bench lab validation; cross-reactivity panel	BTNX Rapid Response™	Manufacturer / CFSRE collab	5 min; competitive	Visual only	Dilutions 0.25–10 µg/mL	None	—	Expanded LOD (~30 analogues); repeatability CV < 15%.

Analytical Accuracy (Paired Data)

Two trials yielded paired data appropriate for diagnostic accuracy assessment. Table 2 summarizes that the CRA (2024) [3] field evaluation encompassed 11 paired street samples validated by GC/MS. Employing a 10 mg/1 mL procedure, the strip demonstrated 100% sensitivity (95% CI 63.1-100) and 100% specificity (95% CI 29.2-100). Upon diluting the identical samples to 10 mg/5 mL, sensitivity decreased to 87.5% (95% CI 47.3-99.7), although specificity persisted at 100%. The sole false negative was observed in the higher-dilution procedure (sample Y4 319). The De Vrieze (2024) [1] laboratory assessment of six genuine powders indicated total agreement with LC-QTOF-MS, yielding six true positives and no false negatives. Due to the limited paired sample size and broad confidence intervals, pooled meta-analysis was not conducted; however, both datasets exhibit exceptionally high accuracy under concentrated conditions (Table 2).

**Table 2:** Analytical accuracy vs MS comparator CRA row Y4 332 excluded (no GC/MS). Exact 95 % CIs use the Clopper–Pearson method. Abbreviations: TP = true positive; FP = false positive; FN = false negative; TN = true negative; CI = confidence interval; Sens = sensitivity; Spec = specificity; GC = gas chromatography; MS: mass spectrometry; CRA = Chicago Recovery Alliance; LC-QTOF-MS = liquid chromatography-quadrupole time-of-flight mass spectrometry

Dataset	Protocol	Unit of analysis	n (paired)	TP	FP	FN	TN	Sensitivity (95 % CI)	Specificity (95 % CI)	Flow/timing	Positivity rule	Reference
CRA (USA) [3]	10 mg/1 mL	Per sample	11	8	0	0	3	100 % (63.1–100)	100 % (29.2–100)	≤ 24 h same aliquot	Faint line = negative	GC/MS (DrugsData)
CRA (USA) [3]	10 mg/5 mL	Per sample	11	7	0	1	3	87.5 % (47.3–99.7)	100 % (29.2–100)	≤ 24 h same aliquot	Faint line = negative	GC/MS (DrugsData)
De Vrieze 2024 [1]	Lab/authentic	Per-sample	6	6	—	0	—	All TP (no FN)	—	≤ 1 h	Faint line = negative	LC-QTOF-MS

Analogue-Level Detection and Cross-Reactivity

Analytical sensitivity showed significant variation among nitazene analogues (Table 3). Isotonitazene demonstrated a limit of detection (LOD) ranging from 2000 to 3000 ng/mL, with uniform cross-reactivity at or below 3000 ng/mL, as reported by De Vrieze (2024) [1], Marland (2025) [2], and Sisco (2024) [5]. Protonitazene necessitated concentrations of 3000-4500 ng/mL, while N-pyrrolidino etonitazene required 1300 ng/mL, although the latter exhibited incomplete solubility in water. N-desethyl metonitazene exhibited the highest sensitivity, identified at approximately 250 ng/mL, while desnitazene derivatives (metodesnitazene, etodesnitazene) sometimes did not respond even at 100 µg/mL. Aggregated findings from investigations revealed that 24 out of 33 nitazene analogues were identified at concentrations of <9000 ng/mL (Table 3).

**Table 3:** Analogue-level limits of detection (LOD) and cross-reactivity Analogue LODs compiled from three lab evaluations (Marland [2], De Vrieze [1], v5). Cross-reactivity indicates any visible test-line loss at ≤ 3000 ng/mL. Abbreviations: LOD = limit of detection; conc = concentration; ESR: Environmental Science and Research, New Zealand

Analogue	Lot ID	Best LOD (ng/mL)	Cross-reactive ≤ 3000 ng/mL?	Read time	Source/notes
Isotonitazene	A & B	2000–3000	Yes	5–10 min	Lot-to-lot variation shown (De Vrieze; v5).
Protonitazene	A	3000–4500	Yes	5 min	Detected consistently (Marland; v5).
N-pyrrolidino etonitazene	A	1300	Yes	5 min	Poor solubility in water (ESR).
N-desethyl metonitazene	A	250	Yes (low LOD)	5 min	Detected easily (Marland).
Metodesnitazene / Etodesnitazene	A	> 100 000	Mixed / often missed	10 min	Detected only at high conc.; lot dependent.
All analogues (aggregate)	—	≤ 9000 for 24/33	—	—	Cross-reactivity confirmed on triplicate runs (De Vrieze; version 5).

Interferences, Matrix Effects, and Operational Findings

Experimental and field data revealed multiple sources of potential influence (Table 4). Caffeine, a prevalent adulterant, resulted in significant attenuation of the test line at around 300 µg/mL, hence posing a danger of false negatives, especially in heroin mixes documented by Marland (2025) [2] and CRA (2024) [3]. Organic solvent concentrations exceeding 10% acetonitrile inhibited adequate wicking, and temperatures surpassing 37 °C diminished line intensity by approximately 10%. Most techniques alleviated these effects by restricting solvent concentration and preserving storage at ambient temperature (15-30 °C). Training in interpretation was advised, highlighting either the manufacturer's guideline (faint line = negative) or a harm-reduction alternative (faint line = positive) to mitigate false reassurance (Table 4).

**Table 4:** Interferences, matrix effects and interpretation Matrix and environmental interference data compiled from lab and manufacturer evaluations; read time = interval from immersion to interpretation. Abbreviations: ACN = acetonitrile; MeOH = methanol; T-line = test line; CRA = Chicago Recovery Alliance; ESR = Environmental Science and Research, New Zealand

Factor	Threshold / effect	Read time	Mitigation	Evidence
Caffeine	Fades T-line ≈ 300 µg/mL → false negative risk	5 min	Use ≤ 5 mg sample / 1 mL protocol	Marland 2025 [2]; CRA [3] heroin mixes.
Organic solvents > 10 % ACN	Wicking failure (no control line)	10 min	Avoid high ACN; prefer ≤ 5 % MeOH	ESR 2024 [4] tech report.
Storage > 37 °C	Band intensity ↓ ~10 %	5 min	Store 15–30 °C	BTNX [3] stability data (v5).
Interpretation rule	Competitive: absence of T = positive	5 min	Train users; optionally “faint line = positive” in harm-reduction	De Vrieze 2024 [1]; Marland 2025 [2].

Risk of Bias and Applicability

The quality assessment with QUADAS-2 is illustrated in Table 5. De Vrieze (2024) [1] exhibited moderate overall risk, chiefly because of the limited sample size and absence of negative controls. Marland (2025) [2] and Sisco (2024) [5] were classified as high risk due to the absence of a reference standard, functioning solely as analytical validations. The CRA (2024) white paper [3] was assessed as high risk due to its manufacturer financing, convenience sampling, and lack of blinded evaluations. Environmental Science and Research (ESR) New Zealand (2024) [4] demonstrated a significant danger for analogous reasons. These restrictions collectively diminish trust in aggregated accuracy estimates while offering significant preliminary evidence regarding test feasibility and performance. Table 5.

**Table 5:** QUADAS-2 risk of bias and applicability QUADAS-2 domains applied to each study. “Flow & timing” judges same-sample testing interval; “Funding bias” added for transparency. Abbreviations: RoB = risk of bias; DTA = diagnostic-test accuracy; MS = mass spectrometry; LC-QTOF = liquid chromatography-quadrupole time-of-flight; LOD = limit of detection

Study	Selection	Index test	Reference standard	Flow & timing	Applicability	Funding bias	Overall RoB	Key limitations
De Vrieze 2024 [1]	Low/Mod.	Low (dual read + ImageJ blinded)	Low (LC-QTOF)	Unclear (no negatives)	Some concerns (lab→field)	Low	Moderate	Small n; no specificity.
Marland 2025 [2]	High (bench panel)	Low	High (no MS)	High	Some concerns	Low	High	Not DTA; only LOD panel.
BTNX/CRA 2024 [3]	High (convenience street sample)	Unclear (field visual)	Moderate (GC/MS)	Unclear	Some concerns	High	High	Gray literature; no blinding.
ESR New Zealand 2024 [4]	High (service report)	Low	High (no MS)	Unclear	Some concerns	Low	High	Not DTA study.
Sisco (2024) [5]	High (manufacturer bench)	Low	High (no MS)	High	Low concern (analytical)	High	High	Analytical validation only.

Discussion 

Summary of Key Findings 

This systematic review presents the inaugural synthesis of analytical performance data for nitazene lateral-flow immunoassay (LFA) test strips in comparison with mass-spectrometry reference methods. Adhering to PRISMA-DTA standards [13] and employing QUADAS-2 [14], the review demonstrates that the LFAs show very good analytical sensitivity under concentrated sample conditions, with reduced sensitivity at higher dilutions. The paired datasets exhibited complete agreement with MS at 10 mg/1 mL [12], decreasing to 87.5% at 10 mg/5 mL, while maintaining 100% specificity. These findings are consistent with established performance patterns in fentanyl test strips (FTS), where analyte concentration, matrix composition, and temperature substantially influence visual thresholds [15]. Similarly, specific excipients (e.g., caffeine ≥300 µg/mL) and solvents (>10% acetonitrile) were observed to affect wicking and diminish line intensity, underscoring the importance of standardized preparation protocols.

Interpretation and Comparison With the Existing Literature

Forensic case reports and toxicological investigations have linked isotonitazene, metonitazene, and etonitazepyne with severe and fatal intoxications [16,17,18,19,20]. Nitazenes represent a structurally diverse class of potent synthetic opioids, distinct from fentanyl but exhibiting comparable or greater μ-opioid receptor activity [21,22]. Detecting this chemically varied class via immunoassay is challenging, as structural heterogeneity influences antibody binding affinity and may result in variable cross-reactivity [23-26].

The dilution-related decline in performance observed in this review aligns with studies [23] and [26], which showed that lower analyte concentrations can reduce line intensity and overall accuracy in fentanyl test strips [23,26,27,28,29]. Studies also highlighted lot- and instruction-dependent variability, underscoring the necessity of rigorous quality-control procedures [27]. Forensic/toxicology evidence-based evaluations similarly demonstrated strong agreement between field LFA testing and mass-spectrometric confirmation when standardized dilution and interpretation procedures were applied [17-20]. From a mechanistic perspective, receptor-binding data and metabolic variability among nitazene analogues [22,23] provide plausible explanations for inconsistent immunoassay reactivity. While LC-MS/MS can achieve robust multi-analyte detection [19], lateral-flow technology will inherently remain constrained by antibody specificity and structural cross-reactivity.

Strengths and Limitations 

A key strength of this review is its methodological rigor, including dual-reviewer screening, structured data extraction, and formal quality assessment using QUADAS-2 [14]. Incorporation of both laboratory-controlled evaluations and field-paired datasets supports a broader applicability of findings. However, the evidence base remains constrained by small paired-sample sizes (n < 20), limited blinding in one study, and incomplete MS verification of negative LFA results. Additional limitations include lot-to-lot variability, non-standardized storage and preparation conditions, and matrix-related interferences. Similar challenges are well documented in prior LFA analytical-accuracy assessments [15,16,27].

Implications for Practice Policy 

These findings bear practical relevance for harm-reduction and forensic screening, where rapid material-based testing can assist in identifying nitazene-containing substances [24,25]. Nitazene LFAs may be used as preliminary screening tools when accompanied by clear interpretive guidance and subsequent confirmatory analysis (e.g., Fourier-transform infrared spectroscopy (FTIR), Raman, or LC-MS) [30,31,32,33,34,35]. Consistent with implementation studies for fentanyl strips [24,25,34,35], accessible and reliable screening methods can support safer decision-making among people who use drugs, such as testing small doses, avoiding solitary use, and carrying naloxone. Given the extreme potency of nitazenes - often exceeding fentanyl by 20-40 fold - the consequences of false-negative screening are substantial. Programs should employ conservative reading protocols (“any absent or faint line = positive”), utilize strict sample-preparation ratios (≤5 mg/1 mL), avoid organic solvents and elevated temperatures, and ensure operator training. Regular lot verification is vital for sustaining analytical reliability [33,34,36]. Integration of nitazene screening into drug-checking systems should emphasize that LFAs supplement - but do not replace - confirmatory laboratory testing [37].

Future Research Directions

Future work should focus on expanding and strengthening the evidence base through large-scale, blinded, multi-site evaluations that incorporate full MS confirmation of both positive and negative samples [38]. Further research is needed to characterize cross-reactivity within the rapidly evolving nitazene family, as structural modifications significantly influence antibody affinity and assay responsiveness [37,38]. Controlled laboratory experiments should explore operational variables - including temperature, dilution ratios, solvent composition, and matrix effects - to define standardized protocols and reference conditions [39]. Comparative assessments across manufacturers and production lots would support the development of formal quality-assurance frameworks for harm-reduction programs [40]. Translational research linking analytical performance to behavioral and overdose-prevention outcomes remains essential [41]. Additionally, the development of multiplex or hybrid screening systems combining LFAs with FTIR, Raman, or LC-MS/MS may provide scalable and contextually relevant approaches to address the ongoing expansion of nitazene distribution [41].

## Conclusions

This systematic review demonstrates that nitrazine lateral-flow immunoassays exhibit promising analytical performance when applied to seized drug materials, particularly under concentrated preparation conditions and standardized operational procedures. Although the strips consistently detected several high-potency nitazene analogues, their performance decreased with greater dilution, variable matrices, and environmental interferences. The evidence base remains limited by small paired sample sizes, incomplete negative controls. 

Despite these limitations, nitazene LFAs may serve as practical preliminary screening tools within harm-reduction and forensic drug-checking systems, provided that results are interpreted conservatively and always confirmed using validated laboratory methods such as LC-MS/MS or GC-MS. To ensure reliability and broader applicability, future work must include large, blinded, multi-site evaluations with full mass-spectrometric confirmation of both positive and negative samples, expanded analogue panels, and systematic assessments of lot variability and operational factors. Improved standardization and analytical validation will be essential for integrating nitrazine LFAs responsibly into drug-monitoring and public-health surveillance frameworks.
